# Iron Overload Damages the Endothelial Mitochondria *via* the ROS/ADMA/DDAHII/eNOS/NO Pathway

**DOI:** 10.1155/2019/2340392

**Published:** 2019-11-12

**Authors:** Huan He, Yang Qiao, Qing Zhou, Zhiqing Wang, Xuepiao Chen, Dan Liu, Dong Yin, Ming He

**Affiliations:** ^1^Jiangxi Provincial Institute of Hypertension, The First Affiliated Hospital of Nanchang University, Nanchang 330006, China; ^2^Jiangxi Provincial Key Laboratory of Basic Pharmacology, Nanchang University School of Pharmaceutical Science, Nanchang 330006, China; ^3^Jiangxi Provincial Key Laboratory of Molecular Medicine, The Second Affiliated Hospital of Nanchang University, Nanchang 330006, China

## Abstract

It has been recognized that iron overload may harm the body's health. Vascular endothelial cells (VECs) are one of the main targets of iron overload injury, and the mechanism involved was thought to be related to the excessive generation of reactive oxygen species (ROS). However, the subcellular and temporal characteristics of ROS generation, potential downstream mechanisms, and target organelles in VECs injured by iron overload have not been expounded yet. In this study, we elucidated the abovementioned issues through both *in vivo* and *in vitro* experiments. Mice were fed pellet diets that were supplemented with iron for 4 consecutive months. Results showed that the thoracic aortic strips' endothelium-dependent dilation was significantly impaired and associated with inflammatory changes, noticeable under brown TUNEL-positive staining in microscopy analysis. In addition, the serum content of asymmetric dimethylarginine (ADMA) increased, whereas nitric oxide (NO) levels decreased. Furthermore, the dimethylarginine dimethylaminohydrolase II (DDAHII) expression and activity, as well as the phosphorylation of endothelial nitric oxide synthase (eNOS) in aortic tissue, were inhibited. Human umbilical vein endothelial cells were treated with 50 *μ*M iron dextran for 48 hours, after which the cell viability, NO content, DDAHII expression and activity, and phosphorylation of eNOS decreased and lactate dehydrogenase and caspase-3 activity, ADMA content, and apoptotic cells significantly increased. After the addition of L-arginine (L-Arg) or pAD/DDAHII, the abovementioned changes were reversed. By dynamically detecting the changes of ROS generation in the cytoplasm and mitochondria and interfering with different aspects of signaling pathways, we have confirmed for the first time that excessive ROS originates from the cytoplasm and activates the ROS-induced ROS release (RIRR) mechanism, leading to mitochondrial dysfunction. Together, our data suggested that excessive free iron ions produced excess ROS in the cytoplasm. Thus, excess ROS create one vicious circle by activating the ADMA/eNOS/DDAHII/NO pathway and another vicious circle by activation of the RIRR mechanism, which, when combined, induce a ROS burst, resulting in mitochondrial dysfunction and damaged VECs.

## 1. Introduction

In recent years, the damage caused by iron overload has attracted increasing attention [1]. Excessive iron intake may cause damage to cells, organs, and even the entire body, thereby leading to variety of diseases, including cardiovascular events. Vascular endothelial cells (VECs) are one of the target cells that are injured by iron overload [2, 3]. Many clinical studies have shown that the patients of hereditary/hemolytic/hemorrhagic diseases or hemodialysis might suffer from VEC injury caused by either iron overload or iron accumulation [4–13]. An iron chelator was effective in clinical treatment [14]. Moreover, iron dextran can cause serious injury in mice [15, 16] or VECs [17–19].

The studies found that oxygen stress or excess intracellular reactive oxygen species (ROS) generation plays a vital role in the pathophysiological processes of iron overload-induced cellular and tissue damage [20–22]. In previous studies, we showed that iron overload can cause excessive ROS generation, resulting in severe liver damage [23, 24]. However, the subcellular and temporal characteristics of ROS generation, potential downstream mechanisms, and target organelles in iron overload-injured VECs have yet to be elucidated.

Latterly, significant attention has been paid to the role of asymmetric dimethylarginine (ADMA) in endothelial dysfunction [25]. Through competition with L-arginine (L-Arg), ADMA inhibits endothelial nitric oxide synthase (eNOS) activity and reduces nitric oxide (NO) production. Dimethylarginine dimethylaminohydrolase (DDAH) contains two isoforms, DDAHI and DDAHII, and metabolizes ADMA. DDAHII is mainly distributed and exerts physiological functions in mammalian VECs [26]. In addition, DDAHII is extremely sensitive to intracellular ROS and decreases in activity, thereby resulting in ADMA accumulation [27]. Clinical studies have shown that plasma ADMA increases in various cardiovascular events, and therefore, ADMA is a cardiovascular risk factor [27, 28]. However, the role of the ADMA/eNOS/DDAHII pathway in iron overload-damaged VECs has not yet been reported.

Oxidative stress and mitochondrial dysfunction have been extensively studied and considered targets of various pathophysiological processes [29]. However, studies on iron overload, VEC damage, and mitochondria are limited [14, 19, 30].

Therefore, the aims of this study were to explore (1) the subcellular and temporal characteristics of ROS generation in iron overload-induced VEC injury, (2) the role of the ADMA/eNOS/DDAHII pathway in iron overload-induced VEC injury, and (3) whether mitochondria are the target organelle of iron overload-induced VEC damage.

## 2. Materials and Methods

### 2.1. Materials, Cells, and Animals

Adenovirus pAD/DDAHII and pAD/DDAHII-shRNA were from GeneChem Co., Ltd. (Shanghai, China). Iron dextran (iron-D), dextran (Dex), phenylephrine (PE), sodium nitroprusside (SNP), acetylcholine (Ach), L-arginine (L-Arg), Edaravone (Eda), N-nitro-l-arginine methyl ester (l-NAME), and ciclosporin A (CsA) were purchased from Sigma-Aldrich (cat. nos. D8517, D9885, P1240000, PHR1423, PHR1546, A5006, M70800, N5751, and C1832, respectively, St. Louis, MO, USA). Antibodies directed against DDAHII, eNOS, eNOS phospho-S1177, cytochrome c (*cyt c*), COX4, and *β*-actin were purchased from Abcam (cat. no. ab1383, ab5589, ab184154, ab16381, ab13575, and ab8229, respectively, Cambridge, UK). Horseradish peroxidase- (HRP-) conjugated IgG was from Jackson ImmunoResearch (cat. no. 107-035-142, West Grove, PA, USA).

Human umbilical vein endothelial cells (HUVECs) were purchased from the China Infrastructure of Cell Line Resources (Shanghai, China). Male C57BL/6J mice, 8-10 weeks old, weighing 20-22 g, were provided by the Animal Center of Nanchang University (Nanchang, China).

All experiments were performed following the National Institutes of Health (NIH) Guidelines for the Care and Use of Laboratory Animals (NIH Publication No. 85-23, revised 1996) and were approved by the Ethics Committee of Nanchang University (Nanchang, China) (No. 2017-0306 (*in vitro*) and 2017-0305 (*in vivo*)).

### 2.2. *In Vivo* Experiments

Mice were housed, two per cage, in a controlled environment at a temperature of 22°C, a humidity of 50%, and a 12-hour light/dark cycle, and water was provided *ad libitum*.

#### 2.2.1. Experimental Grouping *In Vivo*

**Figure 1:**
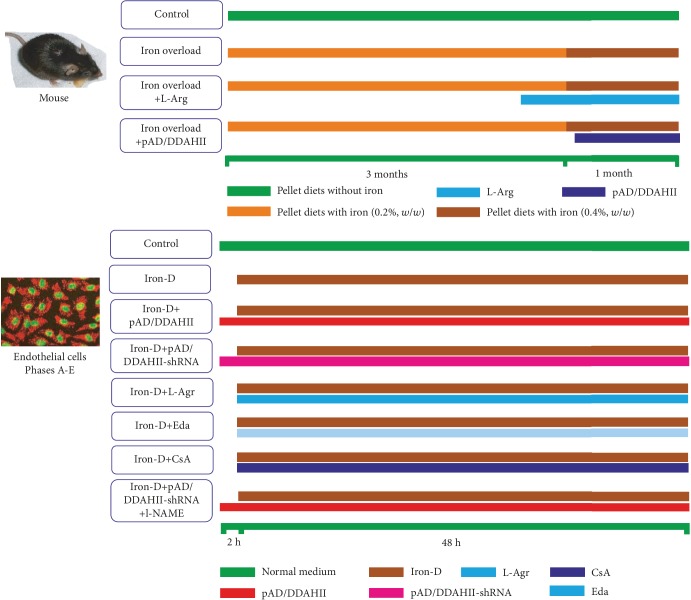
Schematic representation of the experimental design *in vivo* and *in vitro* (see the Sections 2.2.1 and 2.3.2 in the text).

A total of 60 mice were randomly divided into four groups (*n* = 15, Figure 1): three of them were the iron overload group, iron overload+L-Arg group, and iron overload+pAD/DDAHII group, all mice underwent chronic iron overload injury according to our previously published method [31, 32] and were fed a pellet diet for 4 consecutive months (AIN-93G, Medicience Ltd., Yangzhou, China) that was supplemented with iron in the form of ferrocene. The iron content in the diet was maintained at 0.2% (*w*/*w*) for 90 days and then increased to 0.4% (*w*/*w*) for the remaining 30 days. Mice in the iron overload+L-Arg group underwent chronic iron overload for 42 days, and L-Arg (1.5%, pH 6.93) in the drinking water was used as an oral supplementation until the end of the experiment [33]. Mice in the iron overload+pAD/DDAHII group were treated with the similar regime of iron overload for 92 consecutive days; then, pAD/DDAHII adenovirus was injected into the body as follows. The control group consists of mice that were fed a pellet diet (AIN-93G) without iron and had free access to drinking water.

#### 2.2.2. Gene Delivery *via* Tail Vein

A DDAHII overexpression model was constructed in C57BL/6J mice *via* tail vein injection of recombinant adenovirus containing the gene for DDAHII (GenBank ID 23564) as previously described [34]. Briefly, pAD/DDAHII adenovirus (2 × 10^11^ plaque-forming units/ml, 200 *μ*l) was injected via the tail vein. At 4 weeks postinjection, animals were sacrificed.

#### 2.2.3. Collection of Blood and Tissue

At the end of the experiment, animals were weighed and anesthetized using an intraperitoneal injection with ketamine (100 mg/kg) and xylazine (8 mg/kg). Then, blood was collected by cardiac puncture into heparinized capillary tubes and immediately centrifuged for 10 min at 3000 rpm for serum separation. Thoracic aorta rings were harvested in ice-cold physiologic saline solution (PSS: 0.288 g NaH_2_PO_4_, 1.802 g glucose, 0.44 g sodium pyruvate, 20.0 g BSA, 21.48 g NaCl, 0.875 g KCl, 0.7195 g MgSO_4_·7H_2_O, 13.9 g MOPS sodium salt, and 0.185 g EDTA per liter solution at pH 7.4) and evaluated for vascular reactivity as described [34].

#### 2.2.4. Determination of Biochemical and Tissue Injury Indexes

As mentioned previously [32], the iron concentration in serum was determined according to the generation of an iron-ferrozine complex. As a biomarker for tissue injury, the activities of both serum aspartate aminotransferase (AST) and alanine aminotransferase (ALT) were measured by an autoanalyzer (Cobas Integra 400, Roche, Holliston, MA, USA) and an ALT/AST reagent kit from Roche Diagnostics (Indianapolis, IN, USA).

#### 2.2.5. Vascular Reactivity

Vascular contractility and relaxation were determined as previously described [34, 35]. Briefly, thoracic aortas were placed in pressure myograph chambers (DMT Inc., Atlanta, GA, USA), containing warm PSS, cannulated and secured onto glass micropipettes, and equilibrated at an intraluminal pressure of 50 mmHg for 1 hour at 37°C. First, we confirmed that arteries maintained constriction to phenylephrine (PE: 10^−10^-10^−4^ M) for the duration of experiment until no spontaneous dilatation occurred during the constriction period (i.e., 5-12 min). Then, samples were constricted by increasing doses of PE (10^−6^ M, about EC_50_), immediately followed by a dose-response with the endothelium-dependent dilator acetylcholine (ACh: 10^−9^-10^−4^ M). After a washout period and after preconstriction to PE (10^−6^ M), a dose-response to the endothelium-independent dilator sodium nitroprusside (SNP: 10^−10^-10^−4^ M) was evaluated. The percent of dilation was calculated based on the maximal luminal diameter of each artery.

#### 2.2.6. Hematoxylin-Eosin Staining and TUNEL Assay

Freshly harvested thoracic aortas were fixed in 10% buffered formalin solution, embedded in paraffin, and sectioned into 5 *μ*m thick sections that were mounted onto glass slides. To evaluate morphological changes, hematoxylin-eosin (H&E) staining was performed. In addition, to detect apoptosis, the terminal deoxynucleotidyl transferase mediated nick end labelling (TUNEL, Promega, Madison, WI, USA) staining method was performed according to the manufacturer's guidelines [31].

#### 2.2.7. Determination of ADMA and NO Contents

The ADMA content in the serum or culture medium was measured by high-performance liquid chromatography (HPLC) as described previously [36]. Briefly, HPLC was carried out using Agilent 1100 HPLC Systems (Agilent Technologies, Santa Clara, CA, USA) using a ChemStation Edition Workstation and G1313A Autosampler. o-Phthaldialdehyde adducts of methylated amino acids and an internal standard ADMA produced by precolumn mixing were monitored using a model G1321A Fluorescence Detector set at 338 nm (excitation wavelength) and 425 nm (emission wavelength) on Agilent XDB-C18 (50 × 4.6 mm, 1.8 *μ*m). The ADMA content was presented as the amount of ADAM (*μ*mol) per gram protein of serum or per liter of culture medium.

The NO content in serum or the culture medium was indirectly reflected by the contents of nitrite and nitrate [36]. Nitrate is converted to nitrite by aspergillus nitrite reductase, and the total level of nitrite was measured using the Griess reagent (G4410, Sigma-Aldrich, St. Louis, MO, USA), for which the absorbance was determined at 540 nm. The NO content in samples was presented as the amounts of nitrite and nitrate (*μ*M) per gram protein of serum or per liter of culture medium.

#### 2.2.8. Measurement of the Activities of DDAHII

DDAHII activity was measured as described previously [36]. First, the aortic tissue homogenate or lysate of HUVECs (100 *μ*l) was incubated with the work solution (4 mmol/l ADMA and 0.1 mol/l sodium phosphate buffer, 400 *μ*l) for 2 hours at 37°C. The reaction was stopped by the addition of an equal volume of 10% trichloroacetic acid, and the supernatant was boiled with diacetyl monoxime (0.8% (*w*/*v*) in 5% acetic acid) and antipyrine (0.5% (*w*/*v*) in 50% sulfuric acid). Next, the amount of L-citrulline that was formed was determined spectrophotometrically at 466 nm. DDAHII activity was expressed as the amount of L-citrulline (*μ*mol) per min per gram protein of aortic tissue homogenate or HUVEC lysate.

#### 2.2.9. Western Blot Analysis

The total amount of protein from thoracic aorta samples (*in vivo*) and the total amount of mitochondrial proteins from HUVECs (*in vitro*) were extracted using a protein extraction kit (Applygen Technologies Inc., Beijing, China) and a mitochondria isolation kit (Abcam, Cambridge, UK), respectively. A total of 50 *μ*g of protein was separated by denaturing SDS-polyacrylamide gel electrophoresis and transferred to polyvinylidene fluoride membranes. Membranes were then blocked with 5% skim milk, washed, incubated with primary antibodies directed against DDAHII (1 : 1000), eNOS (1 : 1000), eNOS phospho-S1177 (1: 1000), *cyt* C (1 : 1000), *β*-actin (1 : 2000), and COX4 (1 : 1000), and then incubated with an HRP-conjugated secondary antibody. Subsequently, membranes were incubated with an enhanced chemiluminescence reagent for 2 min at room temperature, and protein bands were visualized using an enhanced chemiluminescence method and analyzed with Quantity One software (Bio-Rad, Hercules, CA, USA) [37].

### 2.3. Experiments *In Vitro*

#### 2.3.1. Endothelial Cell Culture and Adenovirus Transfection

For transfection assays, HUVECs were cultivated in high-glucose Dulbecco's modified Eagle's medium (DMEM, Gibco-BRL, Grand Island, NY, USA) supplemented with 10% heat-inactivated fetal bovine serum (FBS, Gibco-BRL, Grand Island, NY, USA), penicillin (100 U/ml), and streptomycin (100 *μ*g/ml) and cultured at 37°C in a humidified atmosphere at 5% CO_2_.

Constructs of pAD/DDAHII (GenBank ID 23564) or pAD/DDAHII-shRNA (GenBank ID 23564, target sequence: gcuccgaauuguggaaauatt) were transfected into HUVECs that were cultured in fresh DMEM supplemented with 15% FBS. For both constructs, the transfection efficiency was roughly 85% after 48 hours. Transfected cells were incubated at 37°C and 95% O_2_ and 5% CO_2_ for 2 hours before being used for experiments.

#### 2.3.2. Experimental Design (Figure 1)

*(1) Phase A*. First, we investigated whether iron overload could induce VEC injury. In addition, we identified the optimal concentration of iron damage that was used for subsequent experiments.

HUVECs were randomly divided into nine groups. Cells in the control group were cultured under normal conditions (37°C, 95% O_2_ and 5% CO_2_) during the entire experiment. Cells in the iron groups were treated with 12.5, 25, 50, 100, or 200 *μ*M iron-D for 48 hours, respectively. To exclude the influence of dextran osmotic pressure, cells in the Dex groups were synchronously treated with 12.5, 25, 50, 100, or 200 *μ*M Dex for 48 hours. At the end of experiments, cell viability and the activity of lactate dehydrogenase (LDH) were determined.

*(2) Phase B*. Next, we confirmed whether iron overload could induce HUVEC injury using various approaches, including multiple phenotypes.

In brief, HUVECs were randomly divided into four groups. HUVECs in the control group were treated as mentioned above. HUVECs in the iron group were treated with 50 *μ*M iron-D for 48 hours, whereas HUVECs in the DDAHII^(+)^ group were treated with pAD/DDAHII for 2 hours before iron-D treatment. HUVECs in the L-Arg group were treated similar to cells in the iron group, but were coincubated with 1 mM L-Arg for 48 hours [38]. At the end of experiments, cell viability, LDH and caspase-3 activities, and apoptosis of HUVECs were determined.

*(3) Phase C*. Next, we investigated whether iron overload in HUVECs could induce an excess in intracellular ROS generation. Furthermore, the subcellular and temporal characteristics of ROS generation were determined and the “ROS-induced ROS release (RIRR)” was investigated.

HUVECs were randomly divided into five groups. Thereinto, control, iron, and L-Arg groups were treated with the above (Phase B). HUVECs in the Eda group or CsA group were treated similar to those in the iron group, and in addition, cells were coincubated with 100 *μ*M Eda or 1 *μ*M CsA for 48 hours, respectively [39, 40]. At 4, 8, 16, 24, and 48 hours after the addition of iron-D, intracellular ROS generation and mitochondrial ROS generation of cells in each group were determined, respectively. At the end of experiments, the cell viability and LDH activity were determined.

*(4) Phase D*. We further investigated how excessive ROS generation impairs VECs, and we explored the ADMA/DDAHII/eNOS/NO signaling pathway.

In brief, HUVECs were randomly divided into six groups. Thereinto, HUVECs in the control, iron, DDAHII^(+)^, and L-Arg groups were treated with the above (Phase B). HUVECs in the DDAHII^(-)^ group were treated with pAD/DDAHII-shRNA for 2 hours before iron-D treatment. Moreover, HUVECs in the DDAHII^(+)^+l-NAME group were treated similar to cells in the DDAHII^(+)^group, and in addition, HUVECs were coincubated for 48 hours with 10 *μ*M l-NAME [41].

At the end of the experiments, cell viability and LDH activity, levels of ADMA and NO in culture medium, expression of eNOS, eNOS phospho-S1177, and DDAHII in the lysate of HUVECs, and DDAHII activity in the lysate of HUVECs were determined.

*(5) Phase E*. Last, we investigated the iron overload damage-inducing effector, mitochondria, as to how it causes dysfunction.

In brief, HUVECs were randomly divided into five groups, namely, control, iron, DDAHII^(+)^, L-Arg, and CsA groups, which were treated with the above (Phase B and Phase C, respectively). At the end of the experiments, the oxygen consumption rate (OCR), extracellular acidification rate (ECAR), mitochondrial membrane potential (MMP), mPTP opening, and *cyt c* release from mitochondria to the cytoplasm in HUVECs were determined.

#### 2.3.3. MTS Assay

HUVECs were plated in 96-well plates at a density of 1 × 10^4^ cells/well and incubated at 37°C with 20 *μ*l MTS (5 mg/ml, Promega, Madison, WI, USA) in 100 *μ*l of DMEM for 2 hours. Next, the absorbance of each well was measured at 490 nm with a microplate reader (Bio-Rad680, Hercules, CA, USA). The absorbance was directly proportional to the number of live cells.

#### 2.3.4. Measurement of LDH Activity

In HUVECs, LDH is an intracellular enzyme that is released into the culture medium upon cell damage [42]. In this study, at the end of the experiment, supernatant was collected and LDH activity was determined with a microplate reader (Bio-Rad680) according to the specifications of the LDH assay kit (Jiancheng, Nanjing, China).

#### 2.3.5. Caspase-3 Activity Assay

Caspase-3 activity was measured in the cytosolic fraction of isolated HUVECs as described previously [31]. Briefly, caspase-3 activity was determined by measuring the cleavage of a caspase-3-specific substrate (acetyl-Asp-Glu-Val-Asp(DEVD)-p-nitroanilide (pNA)(DEVD-pNA)) using a caspase-3 activity assay kit (R&D Systems, Minneapolis, MN, USA) according to the manufacturer's instructions.

#### 2.3.6. Assessment of Endothelial Apoptosis Using Annexin V-FITC and PI

Assessment of apoptosis of HUVECs was performed using an annexin V-EGFP/PI apoptosis detection kit (BD Biosciences, San Diego, CA, USA). Annexin V-stained cells were analyzed using a Cytomics FC500 flow cytometer (Beckman Coulter, Brea, CA, USA), and DCF fluorescence was determined, which is an index of cellular damage [37].

#### 2.3.7. Measurement of Intracellular and Mitochondrial ROS

Levels of intracellular and mitochondrial ROS were measured using a DCFH-DA or mitoSOX probe as described in the previous method [43]. In brief, at 4, 8, 16, 24, and 48 hours after corresponding treatment, cells were harvested, collected, and washed with serum-free DMEM. Then, cells were mixed with serum-free media containing 10 *μ*M DCFH-DA probe (Molecular Probes, Eugene, OR, USA) or 5 *μ*M mitoSOX probe (Thermo Fisher Scientific, Waltham, MA, USA) and incubated at 37°C in the dark for 30 min with slight agitation every 5 min. Subsequently, cell pellets were collected, washed three times with PBS, and resuspended in 500 *μ*l PBS for flow cytometry analysis (Cytomics FC500). The induced green fluorescence from 10,000 cells was documented at 488 or 510 nm. FlowJ software was used to analyze the average fluorescence intensity.

#### 2.3.8. Evaluation of OCR and ECAR

Mitochondrial respiration is an indicator of both the functional bioenergetics capacity of mitochondria and overall cellular health [43, 44]. In our study, we used an XFp Extracellular Flux Analyzer (Seahorse Biosciences, North Billerica, MA, USA) to evaluate the OCR, which was measured as a function of time. In brief, HUVECs were seeded in Seahorse XFp Cell Culture Miniplates at a density of 5000 cells/well and subjected to corresponding treatment. After measurement of basal respiration, oligomycin (complex V inhibitor, 10 *μ*M), carbonyl cyanide-4-(trifluoromethoxy)phenylhydrazone (FCCP, permeabilizing the inner mitochondrial membrane permeable for protons, 2 *μ*M), and rotenone/antimycin A (inhibitors of complex I and III, 0.5 *μ*M/0.5 *μ*M) were added sequentially. The OCR was normalized for total protein per well and expressed as pmol/min.

ECAR was determined by monitoring glycolytic function and was expressed as mpH/min. The measurement procedure was similar to the measurement of OCR described above. After measurement of basal ECAR, glucose solution (80 mM), oligomycin (5 mM), and 2-DG (100 mM) were added sequentially to determine glycolysis, glycolytic capacity, and the glycolytic reserve [44].

#### 2.3.9. Assessment of MMP

Flow cytometry analysis was used to assess the loss of MMP by the fluorescent indicator JC-1 (Invitrogen, Carlsbad, CA, USA). HUVECs were harvested, and the cell suspension was incubated with JC-1 (200 *μ*M) at 37°C for 20 min followed by washing twice with PBS to remove the remaining reagents. Next, fluorescence was measured by Cytomics FC500 flow cytometers with an initial excitation and emission wavelength (ex/me) at 530 and 580 nm (red), followed by ex/em at 485/530 nm (green), respectively. The ratio of red to green fluorescence intensity of cells reflected the level of MMP [37].

#### 2.3.10. Opening of mPTP

Mitochondria of HUVECs were isolated using a mitochondrial/cytosolic fractionation kit (Abcam, Cambridge, UK), resuspended in swelling buffer (KCl 120 mM, Tris-HCl 10 mM, MOPS 20 mM, and KH_2_PO_4_ 5 mM), and plated to a 96-well microtiter plate. 40 *μ*l of CaCl_2_ solution (200 nM) added to each well acted as a stimulant of the opening of the mPTP, and the addition of the solution resulted in a stable decline in mitochondrial density. The absorbance at 520 nm was measured every minute until stable values were observed. To measure the extent of mPTP opening, the changes in absorbance were calculated [42].

### 2.4. Statistical Analysis

All values were expressed as the means ± SEM. Using Origin 8.6 data analysis (OriginLab, Northampton, MA, USA), one-way ANOVA was employed to test the significance of differences in the biochemical data across groups, followed by post hoc testing for individual differences with the Student-Newman-Keuls test. The results were considered significant at a value of *P* < 0.05.

## 3. Results

### 3.1. Changes of General Characteristics, Vascular Responsiveness, Histopathology, and Apoptosis in Iron Overload Mice

General characteristics of mice are shown in Table 1. As expected, the serum iron concentration of all iron intervention mice was significantly higher when compared to that of control mice (*P* < 0.01). Body weight gain in the iron overload group was significantly lower when compared to that in the other three groups (*P* < 0.01). The activities of serum ALT and AST in the iron overload group were significantly higher than those in the control group, but they were significantly improved by L-Arg and pAD/DDAHII treatment (*P* < 0.01). Histological examination confirmed the iron overload-induced tissue and organ damage in mice. In the liver, heart, and islet tissue from iron overload mice, a large amount of iron particles, inflammatory infiltration, spotty necrosis, piecemeal necrosis, and hypertrophy of interstitial cells was observed (see Supplementary Materials, [Supplementary-material supplementary-material-1]).

**Figure 2:**
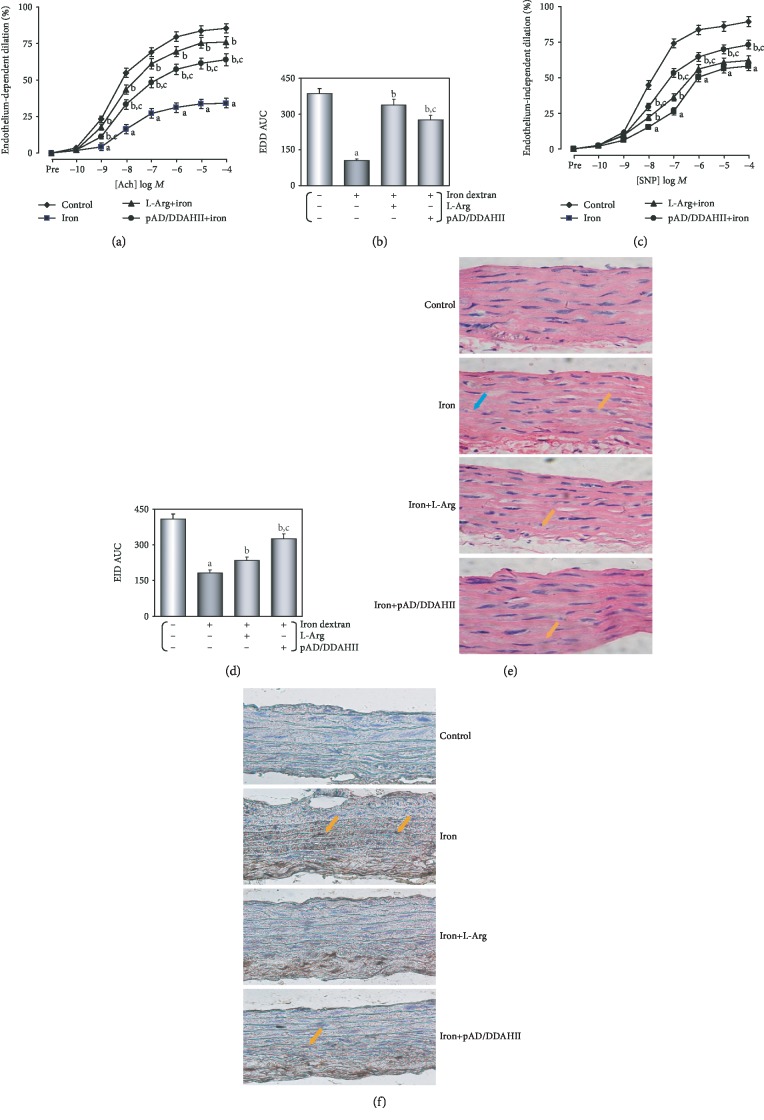
Changes of vascular responsiveness, histopathology, and apoptosis in iron overload mice. (a) Endothelium-dependent dilation (EDD) of thoracic aortic strips. (b) Area under the curve for EDD of thoracic aortic strips. (c) Endothelium-independent dilation (EID) of thoracic aortic strips. (d) Area under the curve for EID of thoracic aortic strips. (e) H&E staining was performed for morphological analysis in the thoracic aorta tissue. Blue arrow: spotty necrosis; orange arrow: hypertrophy of interstitial cells. (f) TUNEL staining was performed for morphological analysis in the thoracic aorta tissue. Orange arrow: TUNEL-positive cells. Data are presented as the mean ± SEM for fifteen individual experiments. ^a^*P* < 0.01 vs. control group; ^b^*P* < 0.01 vs. iron overload group; ^c^*P* < 0.01 vs. iron overload+L-Arg group.

As shown in Figures 2(a) and 2(b), endothelium-dependent dilation (EDD) in the iron overload group was markedly impaired when compared to that in the control group (*P* < 0.01), and the area under the curve (AUC) of the dose-effect relationship was only 27.5% of the control group (*P* < 0.01). Treatment with L-Arg and pAD/DDAHII improved EDD such that dilation was modestly and significantly increased at several doses of Ach (*P* < 0.01). The AUC was more than doubled (*P* < 0.01). Moreover, treatment with L-Arg was almost completely alleviated (87.6%, *P* < 0.01), and pAD/DDAHII treatment was slightly weaker when compared to that of the L-Arg group (71.5%, *P* < 0.01). Similarly, endothelium-independent dilation (EID) in the iron overload group was significantly impaired (*P* < 0.01) and the AUC was 39.7% in the control group (*P* < 0.01, Figures 2(c) and 2(d)). Furthermore, treatment with pAD/DDAHII markedly improved the EID such that the AUC and dilation to most doses of SNP were significantly increased (79.9%, *P* < 0.01); however, L-Arg treatment was slightly weaker (57.4%, *P* < 0.01). Constriction responses to phenylephrine (PE) did not differ among any groups (see Supplementary Materials, [Supplementary-material supplementary-material-1]).

As shown in Figure 2(e), in the iron overload group, inflammatory changes, such as inflammatory infiltration, cell swelling, and interstitial cell hypertrophy, were observed in the vascular endothelium, subendothelial layer, and smooth muscle layer of the thoracic aorta. However, tissue injury was markedly improved in both the iron+L-Arg and iron+pAD/DDAHII groups6. Furthermore, apoptosis of thoracic aorta tissue was assayed using TUNEL staining (Figure 2(f)). Microscopic examination showed that T-positive brown granules in thoracic aorta tissue of the iron overload group were more obvious than those of the control group, indicating that apoptosis of the former was obvious. However, the T-positive brown granules in the thoracic aorta tissues of the iron+L-Arg and iron+pAD/DDAHII groups were obviously alleviated.

### 3.2. Changes of the ADMA/DDAHII/eNOS/NO Pathway in Iron Overload Mice and Its Significance

**Figure 3:**
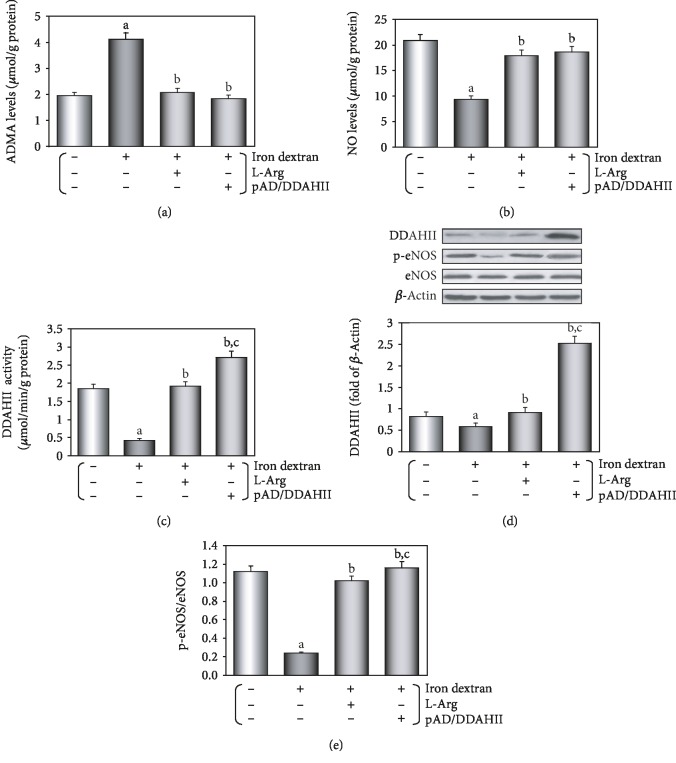
Changes of the ADMA/DDAHII/eNOS/NO signaling pathway in serum and aortic tissue homogenate of iron overload mice. (a) Serum content of ADMA. (b) Serum content of NO. (c) DDAHII expression in aortic tissue. (d) DDAHII activity in aortic tissue. (e) Phosphorylation of eNOS in aortic tissue. (c, e) From left to right: lane 1: control; lane 2: iron; lane 3: iron+L-Arg; lane 4: iron+pAD/DDAHII. Data are presented as the mean ± SEM for ten individual experiments. ^a^*P* < 0.01 vs. control group; ^b^*P* < 0.01 vs. iron overload group; ^c^*P* < 0.01 vs. iron overload+L-Arg group.

As illustrated in Figure 3(a), the serum content of ADMA in iron overload mice was much higher when compared to that in the control group (*P* < 0.01). This change was almost completely counteracted by treatment with L-Arg or iron+pAD/DDAHII (*P* < 0.01). On the contrary, the serum content of NO in iron overload mice was much lower when compared to that in the control group (Figure 3(b), *P* < 0.01). This can almost be completely reversed by L-Arg or pAD/DDAHII treatment (*P* < 0.01).

As shown in Figure 3(c), aortic tissue of mice in the iron overload group showed that DDAHII expression was slightly downregulated (*P* < 0.05) and also slightly upregulated by L-Arg treatment (*P* < 0.01). DDAHII expression was significantly upregulated by pAD/DDAHII treatment (*P* < 0.01). The results also showed that the changes in DDAHII activity were similar to those in DDAHII expression, except that the DDAHII activity in the iron overload group was more significantly inhibited and also not parallel with DDAHII expression which was downregulated (Figure 3(d), *P* < 0.01).

As presented in Figure 3(e), aortic tissue in the iron overload group showed that the p-eNOS/eNOS ratio was reduced when compared to that of the control group (*P* < 0.01). After treatment with L-Arg or pAD/DDAHII, the changes were almost completely reversed (*P* < 0.01).

### 3.3. Iron Overload Could Damage HUVECs

**Figure 4:**
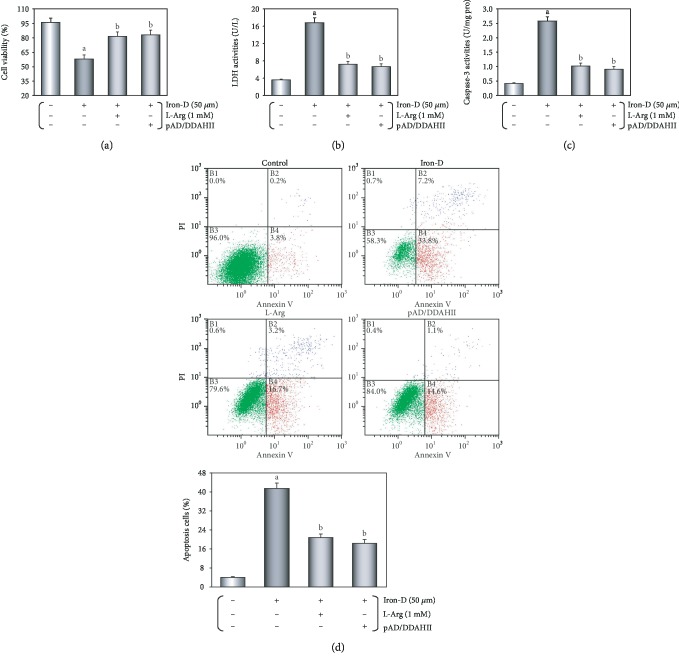
Iron overload-damaged HUVECs. HUVECs were treated with 50 *μ*M iron-D for 48 hours, and iron overload injury was induced. (a) The cell viability of HUVECs. (b) LDH activities in culture media. (c) Histogram of the caspase-3 activity in HUVECs. (d) Flow cytometry dot plots (*x*-axis: annexin V staining, *y*-axis: PI staining) and the quantitation of apoptotic cells. Data are presented as the mean ± SEM for eight individual experiments. ^a^*P* < 0.01 vs. control group; ^b^*P* < 0.01 vs. iron group.

The results of the MTS assay showed that the viability of iron groups in a dose-dependent manner was lower when compared to that of the control group (*P* < 0.01). In addition, the LDH activities of the iron groups were higher when compared to those observed in the control group and were dose-dependent (*P* < 0.01), but cell viability and LDH activity did not change by using Dex of equal concentration gradient, indicating that the changes were the result of iron action and had nothing to do with osmotic pressure (*P* > 0.05, Supplementary Materials, [Supplementary-material supplementary-material-1]). When combining the above two experimental results, the optimal concentration of iron injury was 50 *μ*M, which was selected in the further experiment. As presented in Figures 4(a) and 4(b), after L-Arg and pAD/DDAHII treatment, HUVEC injury was reversed, the cell viability was increased, and the LDH activity in the culture medium was decreased (*P* < 0.01).

However, the cell viability and LDH activity did no change after treatment with L-Arg alone, Eda alone, CsA alone, l-NAME alone, pAD/DDAHII alone, pAD/DDAHII-shRNA alone, and pAD/DDAHII+l-NAME when compared with the control group (*P* > 0.05, Supplementary Materials, [Supplementary-material supplementary-material-1]).

Furthermore, as illustrated in Figure 4(c), the caspase-3 activity in the iron group was significantly increased (*P* < 0.01). However, L-Arg and pAD/DDAHII treatment significantly decreased the caspase-3 activity (*P* < 0.01).

As shown in Figure 4(d), the number of apoptotic cells was notably higher in the iron group (*P* < 0.01). However, treatment with L-Arg and pAD/DDAHII lowered the percentage of apoptotic cells (*P* < 0.01).

### 3.4. Iron Overload in HUVECs Induces Excess Intracellular ROS Generation, and the Role of “ROS-Induced ROS Release”

Our study showed that HUVECs were coincubated with iron-D after the addition of 100 *μ*M Eda [39], a free radical scavenger, and 1 *μ*M CsA [40], a mPTP closing agent, causing the cell viability to increase and the LDH activity in culture medium to decrease (*P* < 0.01, Supplementary Materials, [Supplementary-material supplementary-material-1]).

**Figure 5:**
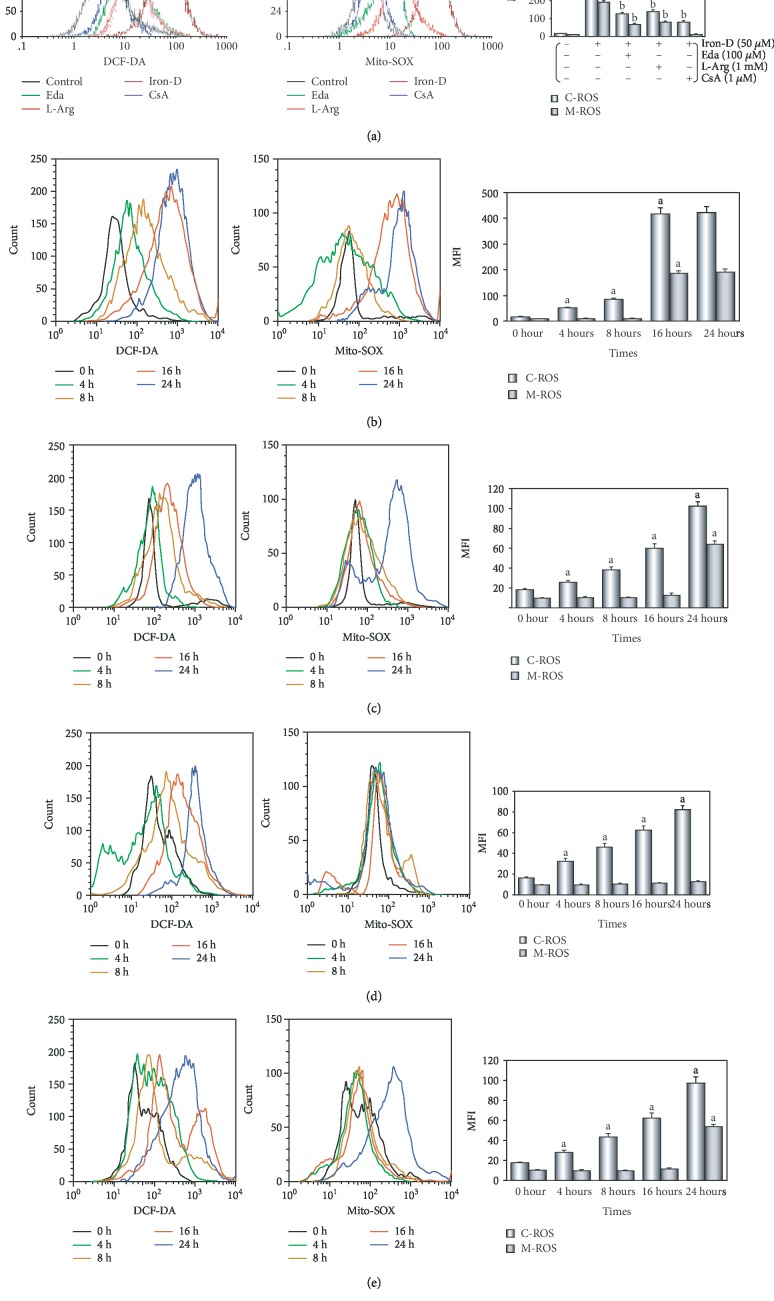
Iron overload in HUVECs induces excess intracellular/mitochondrial ROS generation. (a) After the addition of iron-D for 48 hours, intracellular/mitochondrial ROS generation of HUVECs after different treatments. (b) At various times after adding iron-D, ROS generation of cells in the iron group. (c–e) At various times after adding iron-D, ROS generation of cells in Eda/L-Arg/CsA groups. (b–e) Left: intracellular ROS generation; middle: mitochondrial ROS generation; right: histogram of the intracellular/mitochondrial ROS generation at various times. MFI: mean fluorescence intensity; C-ROS: intracellular ROS; M-ROS: mitochondrial ROS. Data are presented as the mean ± SEM for eight individual experiments. ^a^*P* < 0.01 vs. control group; ^b^*P* < 0.01 vs. iron group.

After the addition of iron-D for 48 hours, the peak of intracellular/mitochondrial ROS levels in HUVECs was significantly moved to the right, thereby indicating a significant increase in the iron group (*P* < 0.01, Figure 5(a)). Moreover, coincubation of HUVECs with Eda/CsA/L-Arg caused a significant shift of the peak of intracellular/mitochondrial ROS in HUVECs to the left, indicating a significant decrease in ROS generation (*P* < 0.01).

As presented in Figure 5(b), in HUVECs, intracellular ROS generation was rapidly and persistently increased (*P* < 0.01) 4 hours after treatment with iron-D, until the burst of ROS generation increased more than 20-fold at 16 hours. Moreover, we found that mitochondrial ROS generation stabilized at baseline between 4 and 8 hours, increased more than 15-fold at 16 hours, and lasted until the end of the experiments at 48 hours.

We found that HUVECs was treated with iron-D after the addition of 100 *μ*M Eda; intracellular ROS generation was slowly and persistently increased with the duration of iron-D treatment, but mitochondrial ROS generation was stable at baseline from the beginning of the experiment to 16 hours (see Figure 5(c)). The time synchronization of ROS burst between the cytoplasm and mitochondria was delayed to 24 hours, and the increase in ROS generation was only 37.2% when treated with iron-D alone. HUVECs was treated with iron-D with the addition of 1 *μ*M CsA; intracellular ROS generation increased slowly and persistently (about 1- to 5-fold) until the end of the 48-hour experiment; however, mitochondrial ROS generation was basically stable at baseline for the duration of iron-D treatment (see Figure 5(e)). In addition, HUVECs were treated with iron-D after the addition of 1 mM L-Arg, an ADMA physiological antagonist; the trend of intracellular ROS and mitochondrial ROS generation was similar to that after the addition of Eda (see Figure 5(d)).

### 3.5. Excessive ROS Generation by Iron Overload Impairs VECs, and the Possible Role of the ADMA/DDAHII/eNOS/NO Pathway

We found that in HUVECs treated with iron-D after the addition of pAD/DDAHII-shRNA or pAD/DDAHII and 10 *μ*M l-NAME, a specific inhibitor of eNOS, the cell viability decreased and the LDH activity in the culture medium increased (*P* < 0.01, Supplementary Materials, [Supplementary-material supplementary-material-1]).

**Figure 6:**
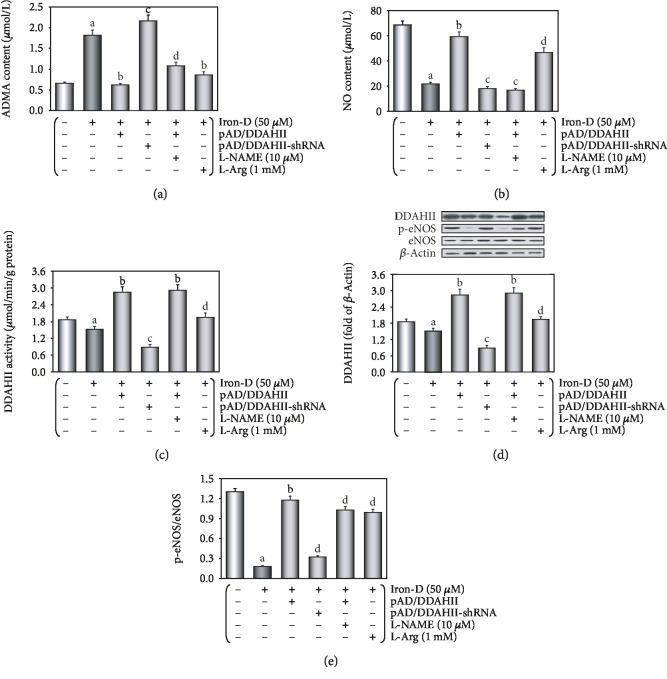
Excessive ROS generation by iron overload impairs HUVECs, and the possible role of the ADMA/DDAHII/eNOS/NO signaling pathway. (a) The content of ADMA in the culture medium. (b) The contents of NO in the culture medium. (c) DDAHII expression in HUVECs. (d) DDAHII activity in HUVECs. (e) Phosphorylation of eNOS in HUVECs. (c–e) From left to right: lane 1: control; lane 2: iron; lane 3: DDAHII^(+)^; lane 4: DDAHII^(-)^; lane 5: DDAHII^(+)^+l-NAME; lane 6: L-Arg. Data are presented as the mean ± SEM for eight individual experiments. ^a^*P* < 0.01 vs. control group; ^b^*P* < 0.01 vs. iron group; ^c^*P* < 0.01 vs. iron group; ^d^*P* < 0.01 vs. DDAHII^(+)^ group.

As presented in Figure 6(a), the content of ADMA in the culture medium was significantly increased following iron overload (*P* < 0.01) and reduced to normal levels in L-Arg- or pAD/DDAHII-treated HUVECs (*P* < 0.01). Cotreatment with pAD/DDAHII-shRNA or pAD/DDAHII and l-NAME reversed this effect again (*P* < 0.01). On the contrary, the NO content in the culture medium was significantly reduced after iron overload (*P* < 0.01) and increased after treatment with L-Arg or pAD/DDAHII. Both pAD/DDAHII-shRNA or pAD/DDAHII and l-NAME significantly decreased the NO content (Figure 6(b), *P* < 0.01).

As shown in Figure 6(c), in an iron overload HUVEC lysate, DDAHII expression was slightly downregulated (*P* < 0.01). Cotreatment with L-Arg or pAD/DDAHII or pAD/DDAHII and l-NAME could upregulate DDAHII expression (*P* < 0.01); however, the effects were reversed by pAD/DDAHII-shRNA (*P* < 0.01). These results showed that the changes of DDAHII activity were similar to those of DDAHII expression, except that DDAHII activity in the iron group was more significantly inhibited. More significantly, in the iron group, the inhibition of DDAHII expression did not correlate with the activity (Figure 6(d), *P* < 0.01).

As presented in Figure 6(e), in an iron overload HUVEC lysate, the p-eNOS/eNOS ratio was reduced (*P* < 0.01). Treatment with both L-Arg and pAD/DDAHII recovered these changes (*P* < 0.01); however, the effects were reversed by pAD/DDAHII-shRNA or pAD/DDAHII and l-NAME (*P* < 0.01).

### 3.6. Iron Overload Damages the Effector Mitochondria and Results in Its Dysfunction

**Figure 7:**
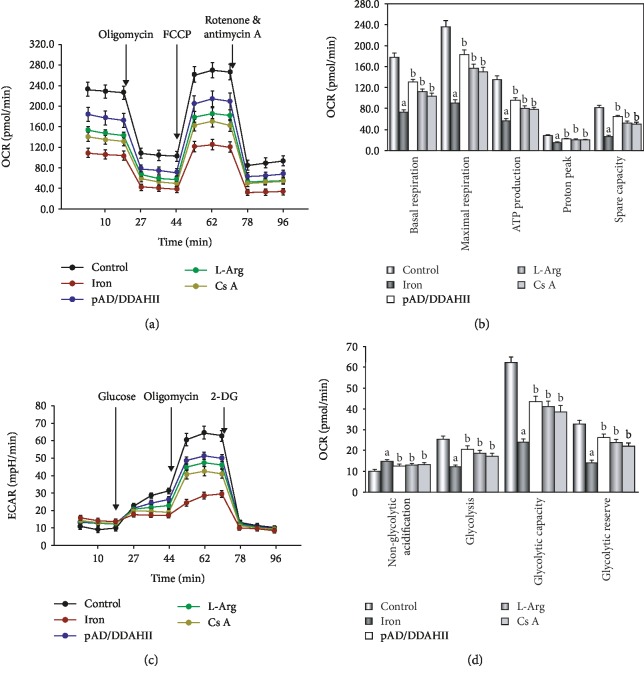
Changes in the oxygen consumption rate and extracellular acidification rate in HUVECs by iron overload injury. (a) OCR curves obtained from HUVECs after different treatments. (b) Histogram of an OCR, an important parameter of HUVECs after different treatments. (c) ECAR curves obtained from HUVECs after different treatments. (d) Histogram of ECAR, an important parameter of HUVECs after different treatments. Data are presented as the mean ± SEM for three individual experiments. ^a^*P* < 0.01 vs. control group; ^b^*P* < 0.01 vs. iron group.

Our data showed that OCR with iron-D treatment was lower when compared to the control group (*P* < 0.01, see Figures 7(a) and 7(b)). Basal respiration, ATP production, proton peak, maximal respiration, and spare respiratory capacity were all significantly lower in HUVECs that were treated with iron-D (*P* < 0.01). Furthermore, with the addition of L-Arg, pAD/DDAHII, or CsA, the abovementioned changes were significantly attenuated (*P* < 0.01).

As presented in Figure 7(c), ECAR of iron-D-treated cells remained lower (*P* < 0.01). In detail, basal rates of glycolysis and glycolytic capacity were significantly lower in HUVECs that underwent iron-D treatment following oligomycin injection (*P* < 0.01). On the contrary, nonglycolytic acidification slightly increased. Similarly, the addition of L-Arg, pAD/DDAHII, or CsA significantly attenuated the abovementioned changes (see Figure 7(d)).

**Figure 8:**
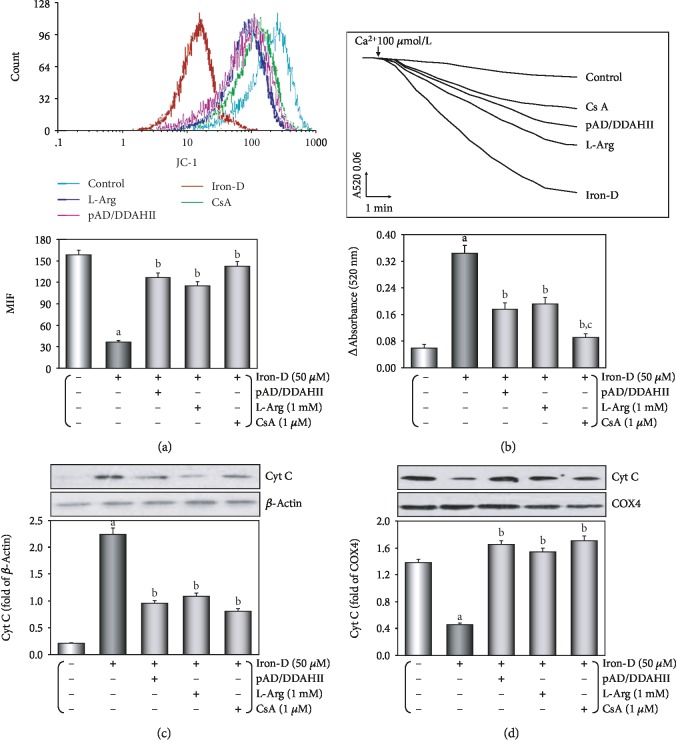
Iron overload induces mitochondrial dysfunction in HUVECs. (a) MMP levels were evaluated by JC-1. The results of the fluorescence peaks on the *x*-axis were used to assess the level of MMP. (b) Ca^2+^-induced swelling of mitochondria was used to determine mPTP opening. The changes in absorbance at 520 nm were recorded every 2 minutes. The data were accessed by the following equation: ΔOD = A520_0 min_ − A520_20 min_. (c) Western blot analysis and histogram of *cyt c* expression in the cytosol. (d) Western blot analysis and histogram of *cyt c* expression in mitochondria. (c, d) From left to right: lane 1: control; lane 2: iron; lane 3: DDAHII^(+)^; lane 4: L-Arg; lane 5: CsA. Data are presented as the mean ± SEM for eight individual experiments. ^a^*P* < 0.01 vs. control group; ^b^*P* < 0.01 vs. iron group.

As shown in Figure 8(a), loss of the MMP occurred after iron-D treatment because the peak of MMP levels significantly shifted to the left (*P* < 0.01). Cotreatment with L-Arg, pAD/DDAHII, or CsA resulted in a significant increase in MMP because of a shift of the peak of MMP to the right (*P* < 0.01).

Figure 8(b) shows that when compared with the control group, after iron-D treatment, the opening of mPTP was triggered (*P* < 0.01). Moreover, in cotreatment with L-Arg, pAD/DDAHII, or CsA, the effect showed a more downward trend when compared to iron-D treatment (*P* < 0.01).

As shown in Figures 8(c) and 8(d), iron-D injury resulted in significant accumulation of *cyt c* in the cytosol (*P* < 0.01), which was significantly reduced when cells were cotreated with L-Arg, pAD/DDAHII, or CsA (*P* < 0.01).

## 4. Discussion

Iron is a necessary trace element for all live animals [1, 2]. It is involved in many important physiological processes, such as electron transport, cell respiration, energy metabolism, and many enzymatic reactions by catalyzing oxidation-reduction reactions [45]. Iron, however, is a double-edged sword. Iron deficiency (anemia) is the most common public nutrition problem in the world [46]. Excess iron also has toxic effects on the body, and iron overload results in various diseases caused by excessive free radicals in the body [1]. In general, the target organs of iron overload injury involve the liver, heart, central nervous system, and islets of the pancreas [20–24].

In recent years, VECs were realized as important target organs of iron overload-induced injury [2, 3]. Many clinical investigations focus on hereditary hematologic diseases [4–10] (such as *β*-thalassemia, sickle cell anemia, and HFE hemochromatosis myelodysplastic syndromes), hemolytic/hemorrhagic diseases [11] (such as hemorrhagic stroke and traumatic brain injury), neurodegenerative disorders [12] (such as Parkinson's and Alzheimer's disease), or treatment by hemodialysis [13], because patients with these diseases are most likely to suffer from iron accumulation or iron overload-induced VEC injury. Iron chelators have been shown to be effective in clinical treatment [14]. Foundational researches have also found that iron dextran can cause significant VEC injury *in vivo* [15, 16] or *in vitro* [17–19]. In this study, we confirmed that iron overload can cause severe damage to VECs. The *in vivo* study showed that mice were fed pellet diets for 4 months, supplemented with iron, the thoracic aortic strips' EDD was significantly impaired, and inflammatory changes were observed by histopathology, which were noticeable as brown TUNEL-positive cells in microscopy (see Figure 2). In our *in vitro* data showing that in HUVECs that were treated with 50 *μ*M iron-D for 48 hours, the cell viability was decreased and the LDH activity, caspase-3 activity, and apoptotic cells significantly increased (see Figure 4). These results were consistent with the mainstream literature reports [15–19].

There is growing evidence that iron overload-induced excessive ROS generation triggers subsequent pathophysiological changes [16–18, 20–22]. In this study, we found that in HUVECs that were treated with iron-D for 48 hours, the intracellular and mitochondrial ROS generation significantly increased (see Figure 5(a)), thereby indicating that increased ROS was responsible for cell damage.

Subsequently, we determined the intracellular and mitochondrial ROS generation at different stages and found that there was a significant difference; that is, mitochondrial ROS generation significantly lagged behind the cytoplasm (see Figure 5(b)). Furthermore, the time (16 hours) was surprisingly consistent in the sharp increase in the intracellular and mitochondrial ROS (ROS burst). This phenomenon was similar to that of the ROS-induced ROS release (RIRR) hypothesis [47]. The hypothesis holds that when ROS generation is increased, the MMP is unstable, leaving the mPTP in a continuous open state. Mitochondrial swelling leads to mitochondrial membrane rupture, thereby irreversibly damaging mitochondria. Consequently, as an important component of the content, ROS is released from the matrix to the cytosol and rapidly taken up by neighboring normal mitochondria, which induced these neighboring mitochondria to alter analogously, thereby ultimately leading to apoptosis [48, 49]. Interestingly, and somewhat more surprisingly, we found that in HUVECs that were treated with iron-D with the addition of Eda, ROS burst was significantly delayed and its intensity was weakened (see Figure 5(c)). These results further confirmed that excessive ROS is produced in the cytoplasm because Eda, a free radical scavenger, can directly destroy free radicals in the cytoplasm similar to endogenous antioxidant enzymes such as DDAHII and SOD. However, when ROS is generated excessively and exceeds the processing capacity of Eda, it may also induce the RIRR mechanism. Similarly, CsA, an mPTP closing agent, closes the mPTP; a significant increase (ROS burst) in ROS generation in both the cytoplasm and the mitochondria for the duration of iron-D treatment disappears, confirming that this phenomenon was due to excessive ROS in the early cytoplasm entering the mitochondria and inducing RIRR (see Figure 5(e)). As a specific mPTP closing agent, even though a small amount of ROS was produced in the cytoplasm, CsA also ensured that it does not cause destructive damage to the cells. These results provided convincing evidence that excessive ROS generation originates in the cytoplasm; RIRR activation plays an important role. Eda inactivates ROS in the cytoplasm and CsA closes mPTP, both of which can inhibit the RIRR mechanism and maintain mitochondrial function, therefore increasing the cell viability and decreasing the LDH activity in the culture medium (see [Supplementary-material supplementary-material-1]). It must be admitted that if antioxidants such as MitoQ, which can be scavenged by targeted mitochondrial ROS, can be selected at this time and if it can cancel the RIRR mechanism and reduce secondary VEC damage and dysfunction, the conclusion of this study will be more solid.

Due to the oxidative properties of iron itself, it reacts with related substances to produce oxygen free radicals, which increase ROS generation in VECs and can be inactivated by the endogenous antioxidant enzyme system in the early stage. However, after excessive ROS generation, it not only inhibits DDAHII activity and ADMA metabolism but also affected NO synthesis. ROS can enter mitochondria, thereby affecting the energy metabolism of mitochondria and the electron transfer of the respiratory chain. This will cause more ROS generation, induce a self-amplifying process, and lead to a ROS burst and mitochondrial dysfunction. Thus, the mPTP plays a crucial role in RIRR [49], and ROS is one of the most important factors for stimulating opening of the mPTP [50] and ultimately results in a vicious circle. At this time, our exogenous supplementation of L-Arg (see Figure 5(d)) or upregulation of DDAHII expression can not only enhance the ability of cells to degrade ADMA but also inhibit ROS generation, effectively alleviating and reducing the damage of iron overload-induced VECs. Through suppressing one or more targets, the vicious circle could be broken. The results showed that Eda and CsA were similar to acute stroke, and their targets might be molecular aims for intervention of iron overload-induced VEC injury [51].

A large number of foundational and clinical studies have confirmed that endothelial dysfunction is an early pathophysiological change in many cardiovascular diseases, including atherosclerosis, coronary heart disease, and hypertension [2, 3, 27, 28]. Endothelial dysfunction is often associated with alterations in the ROS/ADMA/eNOS/DDAHII pathway [25]. As the main metabolic enzyme of ADMA, DDAHII is highly sensitive to intracellular ROS generation, which inhibits its activity, thereby leading to ADMA accumulation [27]. The latter competes with L-Arg to inhibit eNOS activity and reduce the synthesis of NO [26]. However, NO plays an important role in the maintenance of vascular tone and structure [26]. As an independent predictor, ADMA leads to the uncoupling of NOS in VECs and results in further increasing superoxide production, which in turn reduces the bioavailability of NO and leads to endothelial dysfunction [52, 53]. In this study, we first confirmed that the ADMA/eNOS/DDAHII pathway also plays a major role in iron overload-induced VEC injury. Moreover, our *in vivo* study showed that in mice that were fed a pellet diet for 4 months supplemented with iron, the serum contents of ADMA increased and NO decreased and DDAHII expression and activity and phosphorylation of eNOS in aortic tissue were inhibited (see Figure 3). In treatment with L-Arg or pAD/DDAHII, that is, after application of an ADMA competitive substrate or upregulating DDAHII expression, vascular responsiveness, histopathological and apoptotic changes, and other indexes mentioned above in iron overload mice improved (see Figures 2 and 3). In this *in vitro* study, we showed that in HUVECs that were treated with iron-D for 48 hours, the contents of ADMA increased and DDAHII expression and activity, the contents of NO, and phosphorylation of eNOS decreased (see Figure 6). Similarly, in L-Arg or pAD/DDAHII treatments, the cell viability, ROS generation, and other indexes mentioned above in iron overload-induced HUVEC damage were significantly improved. However, cotreatment with pAD/DDAHII-shRNA or pAD/DDAHII adding with l-NAME, an eNOS-specific inhibitor, reversed the changes observed (see Figures 4–6). These results indicated that the ADMA/eNOS/DDAHII pathway may become a molecular target in the treatment of iron overload-induced VEC injury.

Mitochondria are multifunctional organelles and can actively or passively drive cellular dysfunction or demise [54, 55]. Certainly, the structural and functional integrity is fundamental to cellular life. Simultaneously, apoptosis, degeneration, and necrosis often occur in VEC injury [56, 57]. Initial events in mitochondria leading to apoptosis are the permeability transition in the inner membrane leading to outer membrane rupture and permeabilization of the outer membrane, thereby permitting the release of apoptogenic factors, including *cyt c* release from the mitochondrial intermembrane space [52]. Necrosis is characterized by mitochondrial membrane depolarization, decreased ATP levels, cellular and organellar swelling, and loss of integrity of the membrane [54]. Therefore, mitochondria are the target organelles of various pathophysiological processes and may become molecular targets of related disease therapeutics [58, 59]. In this study, we revealed that in HUVECs that were treated with iron-D for 48 hours, mitochondrial function was marked impaired, indicating that mitochondrial respiration and glycolytic function (the abilities of oxidative phosphorylation and ATP production) were weakened significantly (see Figure 7), impeded MMP, mediated mitochondrial swelling, opened mPTP, and released *cyt c* from the mitochondria into the cytosol. Of course, adding L-Arg or pAD/DDAHII or CsA caused the abovementioned mitochondrial function to significantly recover and improve (see Figure 8). These results indicated that mitochondria are the target organelles of iron overload-induced VEC damage.

**Figure 9:**
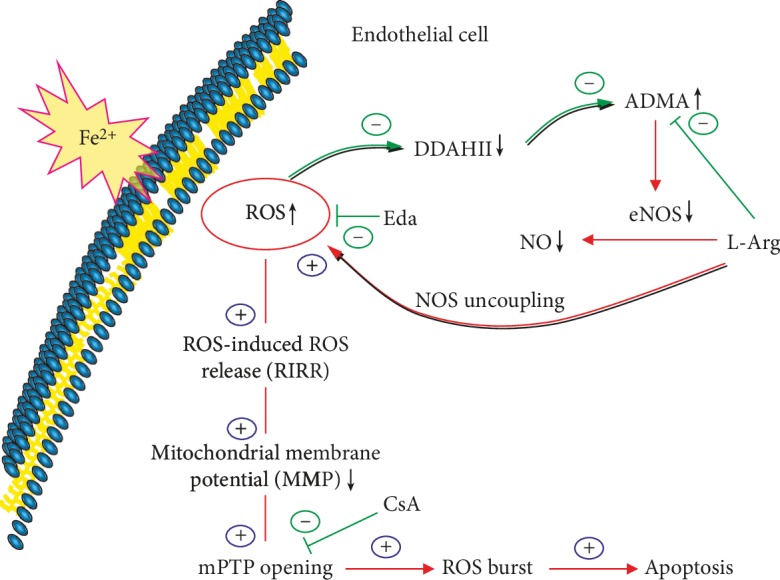
Diagram showing the possible mechanism of iron overload-induced injury to VECs. Excessive free iron ions produce excess ROS in the cytoplasm. The latter results in biological effects in two ways: excess ROS inhibits DDAHII and accumulates ADMA. ADMA not only inhibits eNOS activity competitively and decreases NO synthesis but also induces eNOS uncoupling and produces even more ROS, thereby cycling and reciprocating ROS, forming one vicious cycle. In addition, excess ROS entered mitochondria, weakening the MMP, opening the mPTP, activating the RIRR mechanism, and forming another vicious circle. These two circles together induce ROS burst, leading to mitochondrial dysfunction, which in turn damages VECs.

In summary, we determined ROS as the core and outline the possible mechanism of iron overload-induced VEC injury (see Figure 9). Excessive free iron ions produce excess ROS in the cytoplasm. The latter results in biological effects in two ways: excess ROS inhibits DDAHII and accumulates ADMA. ADMA not only inhibits eNOS activity competitively and decreases NO synthesis but also induces eNOS uncoupling and produces even more ROS, thereby cycling and reciprocating ROS, forming one vicious cycle. In addition, excess ROS entered mitochondria, thereby weakening MMP, opening mPTP, activating the RIRR mechanism, and forming another vicious circle. These two circles together induce ROS burst, leading to mitochondrial dysfunction, which in turn damages VECs. Therefore, interrupting any step of the abovementioned cycles can end the related vicious cycle and prevent the occurrence and development of injury.

## Figures and Tables

**Table 1:** Serum iron concentration, body weight, and serum activities of ALT and AST in each treatment group of mice.

	Control	Iron	Iron+L-Arg	Iron+pAD/DDAHII
Body weight (g)	33.2 ± 1.6	27.1 ± 2.5^a^	31.5 ± 2.1^c^	30.9 ± 1.8^c^
Serum iron concentration (*μ*mol/l)	30.5 ± 1.4	408.6 ± 17.6^a^	382.8 ± 16.2^a,b^	388.5 ± 17.1^a,b^
Serum ALT activity (U/l)	46.2 ± 1.6	245.2 ± 9.3^a^	130.5 ± 5.2^a,c^	170.6 ± 9.3^a,c,d^
Serum AST activity (U/l)	95.8 ± 3.8	388.4 ± 15.3^a^	209.5 ± 9.8^a,c^	262.3 ± 13.2^a,c,d^

Data are expressed as the mean ± SEM (*n* = 15). ^a^*P* < 0.01 vs. control group; ^b^*P* > 0.05 vs. iron group; ^c^*P* < 0.01 vs. iron group; ^d^*P* < 0.01 vs. iron+L-Arg group. ALT: alanine transaminase; AST: aspartate transaminase.

## Data Availability

The data used to support the findings of this study are included within the article.

## Abbreviations

ACh: Acetylcholine
ADMA: Asymmetric dimethylarginine
ALT: Alanine aminotransferase
AST: Aspartate aminotransferase
AUC: Area under the curve
CsA: Ciclosporin A
*cyt c*: Cytochrome C
DCFH-DA: 6-Carboxy-2′-7′-dichlorodihydro-flurescein diacetate
DDAHII: Dimethylarginine dimethylaminohydrolase II
Dex: Dextran
DMEM: Dulbecco's modified Eagle's medium
ECAR: Extracellular acidification rate
Eda: Edaravone
EDD: Endothelium-dependent dilation
EID: Endothelium-independent dilation
eNOS: Endothelial nitric oxide synthase
FBS: Fetal bovine serum
H&E: Hematoxylin-eosin staining
HPLC: High-performance liquid chromatography
HUVECs: Human umbilical vein endothelial cells
L-Arg: L-Arginine
LDH: Lactate dehydrogenase
l-NAME: N-Nitro-l-arginine methyl ester
IHC: Immunohistochemistry
Iron-D: Iron dextran
MDA: Malondialdehyde
MMP: Mitochondrial membrane potential
mPTP: Mitochondrial permeability transition pore
NO: Nitric oxide
OCR: Oxygen consumption rate
PE: Phenylephrine
PSS: Physiologic saline solution
RIRR: ROS-induced ROS release
ROS: Reactive oxygen species
SNP: Sodium nitroprusside
SFN: Sulforaphane
TUNEL: Terminal deoxynucleotidyl transferase dUTP nick-end labeling
VECs: Vascular endothelial cells.

## Supplementary Materials

Supplementary Materials Figure S1: histopathological changes of tissue and organ of mice injured by iron overload. A large amount of iron particle accumulation (pink arrow), inflammatory infiltration (green arrow), spotty necrosis (blue arrow), piecemeal necrosis (yellow arrow), and hypertrophy of interstitial cells (orange arrow) was exhibited (400x). (A) Heart. (B) Liver. (C) Islet tissue. Figure S2: mice's thoracic aorta constriction to phenylephrine (PE: 10^−10^-10^−4^ M). Constriction responses to PE did not differ among any group's thoracic aortas (*P* < 0.05). Data are presented as the mean ± SEM for fifteen individual experiments. Figure S3: effects of iron-D and equal concentration Dex on the cell viability and LDH activity of HUVECs. HUVEC viability/LDH activity with iron-D treatment was lower/higher in a dose-dependent manner, but cell viability and LDH activity did not change by using Dex of equal concentration gradient, indicating that the changes were the result of iron action and had nothing to do with osmotic pressure. (A) Histogram of the cell viability. (B) Histogram of the LDH activity. Data are presented as the mean ± SEM for eight individual experiments. a: *P* < 0.01 vs. prior dosage. Figure S4: effects of Eda/CsA/L-Arg, up-/downregulated DDAHII expression, or upregulated DDAHII expression with the addition of l-NAME on the cell viability and LDH activity of HUVECs. Cell viability and LDH activity did not change by using Eda alone (100 *μ*M), CsA alone (1 *μ*M), L-Arg alone (1 mM), up-/downregulated DDAHII expression alone, or upregulated DDAHII expression with the addition of l-NAME (10 *μ*M) when compared with the control group (*P* < 0.05). (A) Histogram of the cell viability. (B) Histogram of the LDH activity. Data are presented as the mean ± SEM for eight individual experiments. Figure S5: effects of Eda/CsA/L-Arg on the cell viability and LDH activity of HUVECs injured by 50 *μ*M iron-D. Eda/CsA/L-Arg with 50 *μ*M iron-D was used to cotreat HUVECs; the cell viability increased and LDH activity decreased. (A) Histogram of the cell viability. (B) Histogram of the LDH activity. Data are presented as the mean ± SEM for eight individual experiments. a: *P* < 0.01 vs. control group; b: *P* < 0.01 vs. iron group. Figure S6: effects of up-/downregulated DDAHII expression or upregulated DDAHII expression with the addition of l-NAME on the cell viability and LDH activity of HUVECs injured by 50 *μ*M iron-D. Upregulated DDAHII expression protected HUVECs against iron overload injury, but the added l-NAME reversed the effects. Downregulated DDAHII expression could aggravate HUVEC damage induced by iron overload. (A) Histogram of the cell viability. (B) Histogram of the LDH activity. Data are presented as the mean ± SEM for eight individual experiments. a: *P* < 0.01 vs. control group; b: *P* < 0.01 vs. iron group.
