# Supplementary material for: Iron Overload Damages the Endothelial Mitochondria via the ROS/ADMA/DDAHII/eNOS/NO Pathway
Source: Oxid Med Cell Longev. 2019 Nov 12;2019:2340392. doi: 10.1155/2019/2340392 (PMC6875360; doi:10.1155/2019/2340392)
Supplement: Supplementary Materials — Figure S1: histopathological changes of tissue and organ of mice injured by iron overload. A large amount of iron particle accumulation (pink arrow), inflammatory infiltration (green arrow), spotty necrosis (blue arrow), piecemeal necrosis (yellow arrow), and hypertrophy of interstitial cells (orange arrow) was exhibited (400x). (A) Heart. (B) Liver. (C) Islet tissue. Figure S2: mice's thoracic aorta constriction to phenylephrine (PE: 10−10-10−4 M). Constriction responses to PE did not differ among any group's thoracic aortas (P < 0.05). Data are presented as the mean ± SEM for fifteen individual experiments. Figure S3: effects of iron-D and equal concentration Dex on the cell viability and LDH activity of HUVECs. HUVEC viability/LDH activity with iron-D treatment was lower/higher in a dose-dependent manner, but cell viability and LDH activity did not change by using Dex of equal concentration gradient, indicating that the changes were the result of iron action and had nothing to do with osmotic pressure. (A) Histogram of the cell viability. (B) Histogram of the LDH activity. Data are presented as the mean ± SEM for eight individual experiments. a: P < 0.01 vs. prior dosage. Figure S4: effects of Eda/CsA/L-Arg, up-/downregulated DDAHII expression, or upregulated DDAHII expression with the addition of l-NAME on the cell viability and LDH activity of HUVECs. Cell viability and LDH activity did not change by using Eda alone (100 μM), CsA alone (1 μM), L-Arg alone (1 mM), up-/downregulated DDAHII expression alone, or upregulated DDAHII expression with the addition of l-NAME (10 μM) when compared with the control group (P < 0.05). (A) Histogram of the cell viability. (B) Histogram of the LDH activity. Data are presented as the mean ± SEM for eight individual experiments. Figure S5: effects of Eda/CsA/L-Arg on the cell viability and LDH activity of HUVECs injured by 50 μM iron-D. Eda/CsA/L-Arg with 50 μM iron-D was used to cotreat HUVECs; the cell viability increased and [file 2340392.f1.pdf]

Supplementary materials

Figure S1. Histopathological changes of tissue and organ of mice injuried by iron overload. A large amount of iron particles accumulation (Pink arrow), inflammatory infiltration (Green arrow), spotty necrosis (Blue arrow), piecemeal necrosis (Yellow arrow), and hypertrophy of interstitial cells (Orange arrow) were exhibited (400×). (A) Heart. (B) Liver. (C) Islet tissue.

Figure S2. Mice's thoracic aortas constriction to phenylephrine (PE: 10^{-10}-10^{-4} M). Constriction responses to PE did not differ among any group's thoracic aortas (P> 0.05). Data are presented as the mean ± SEM. for fifteen individual experiments.

Figure S3. Effects of iron-D and equal concentration Dex on the cell viability and LDH activity of HUVECs. HUVECs viability/LDH activity of iron-D treated were lower/higher in a dose-dependent manner, but cell viability and LDH activity did not change by using Dex of equal concentration gradient, indicating that the changes were the result of iron action and had nothing to do with osmotic pressure. (A) Histogram of the cell viability. (B) Histogram of the LDH activity. Data are presented as the mean ± SEM. for eight individual experiments. a: P<0.01, *vs* prior dosage.

Figure S4. Effects of Eda/CsA/L-Arg, or up-/downregulated DDAHⅡexpression, or upregulated DDAHⅡexpression added l-NAME on the cell viability and LDH activity of HUVECs. Cell viability and LDH activity did not change by using Eda alone (100 μM), CsA alone (1 μM), L-Arg alone (1 mM), or up-/downregulated DDAHⅡexpression alone, or upregulated DDAHⅡexpression added l-NAME (10 μM) when compared with the control group (p>0.05). (A) Histogram of the cell

viability. (B) Histogram of the LDH activity. Data are presented as the mean ± SEM. for eight individual experiments.

Figure S5. Effects of Eda/CsA/L-Arg on the cell viability and LDH activity of HUVECs injuried by 50 μM iron-D. Eda/CsA/L-Arg with 50 μM iron-D co-treat HUVECs, the cell viability increased and LDH activity decreased. (A) Histogram of the cell viability. (B) Histogram of the LDH activity. Data are presented as the mean ± SEM. for eight individual experiments. a: $p<0.01$, vs. control group; b: $p<0.01$, vs. iron group.

Figure S6. Effects of up-/downregulated DDAHⅡexpression, or upregulated DDAHⅡexpression added l-NAME on the cell viability and LDH activity of HUVECs injuried by 50 μM iron-D. Upregulated DDAHⅡexpression protected HUVECs against iron overload injury, but added l-NAME reversed the effects. Downregulated DDAHⅡexpression could aggravate HUVECs damage induced by iron overload. (A) Histogram of the cell viability. (B) Histogram of the LDH activity. Data are presented as the mean ± SEM. for eight individual experiments. a: $P<0.01$, vs. control group; b: $P<0.01$, vs. iron group.

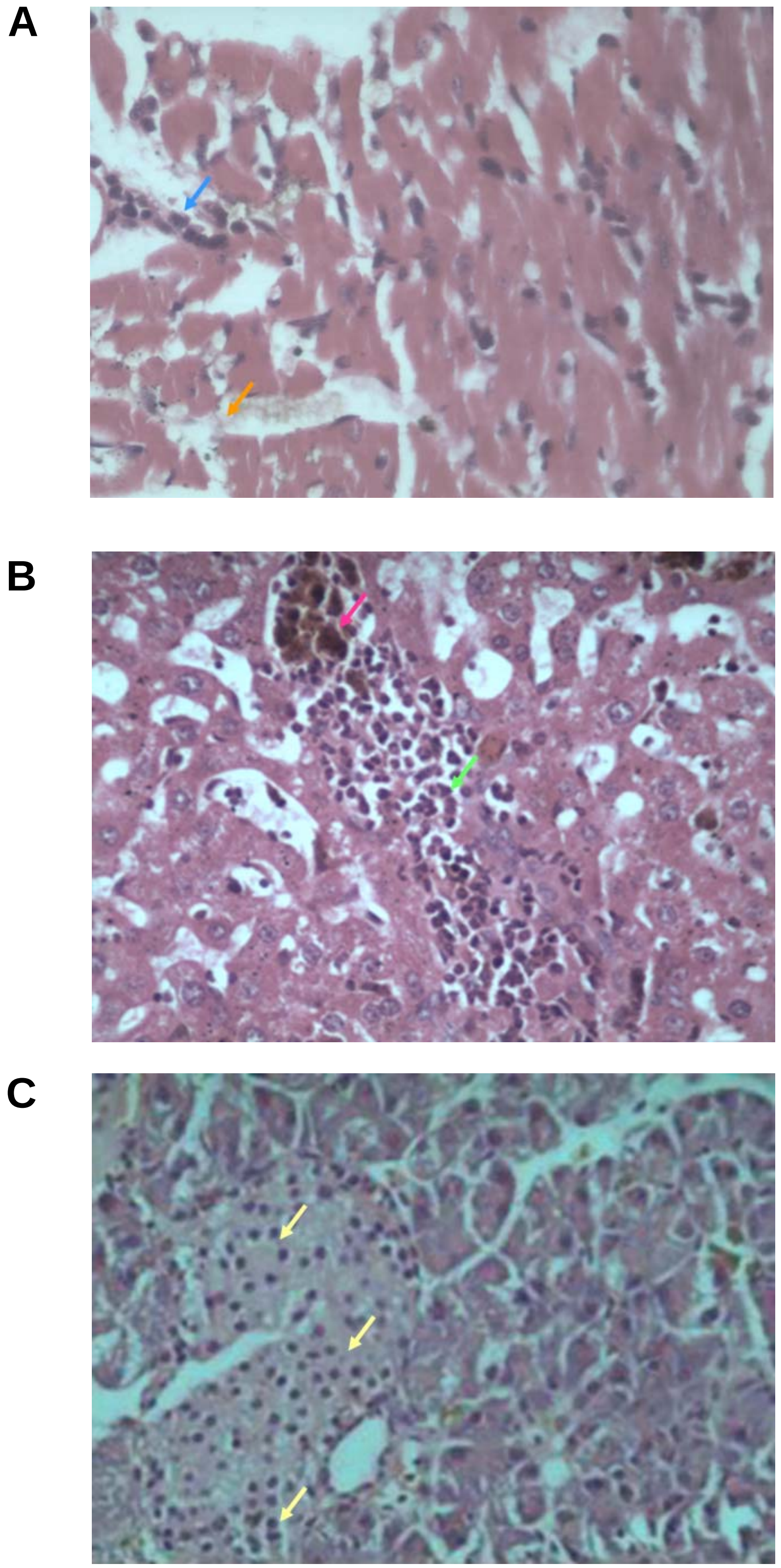

Figure S1

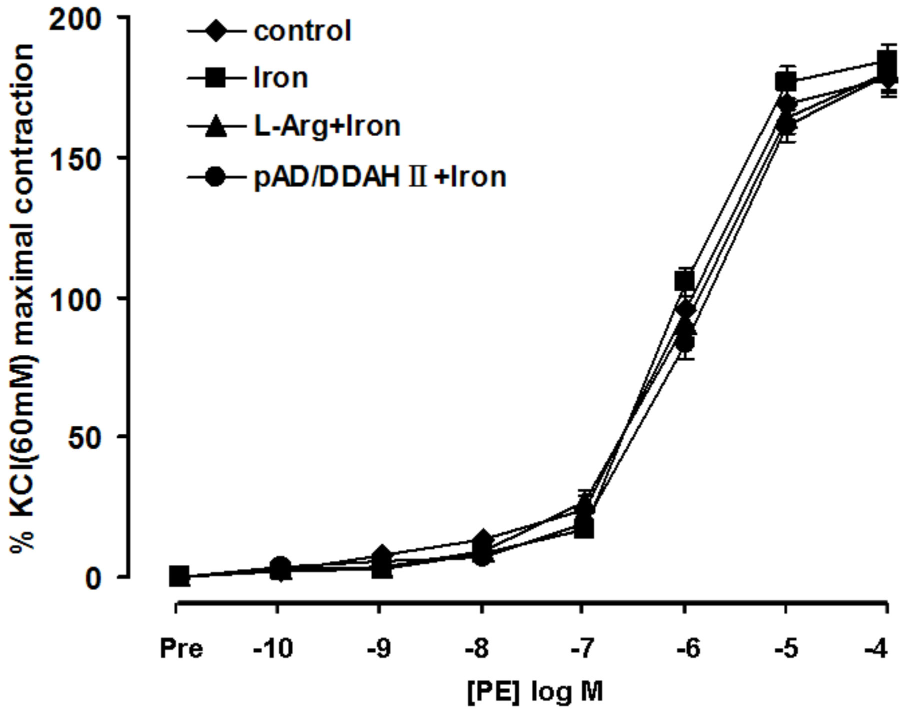
control
Iron
L-Arg+Iron
pAD/DDAH Ⅱ +Iron
% KCl(60mM) maximal contraction
200
150
100
50
0
Pre
-10
-9
-8
-7
-6
-5
-4
[PE] log M

Figure S2

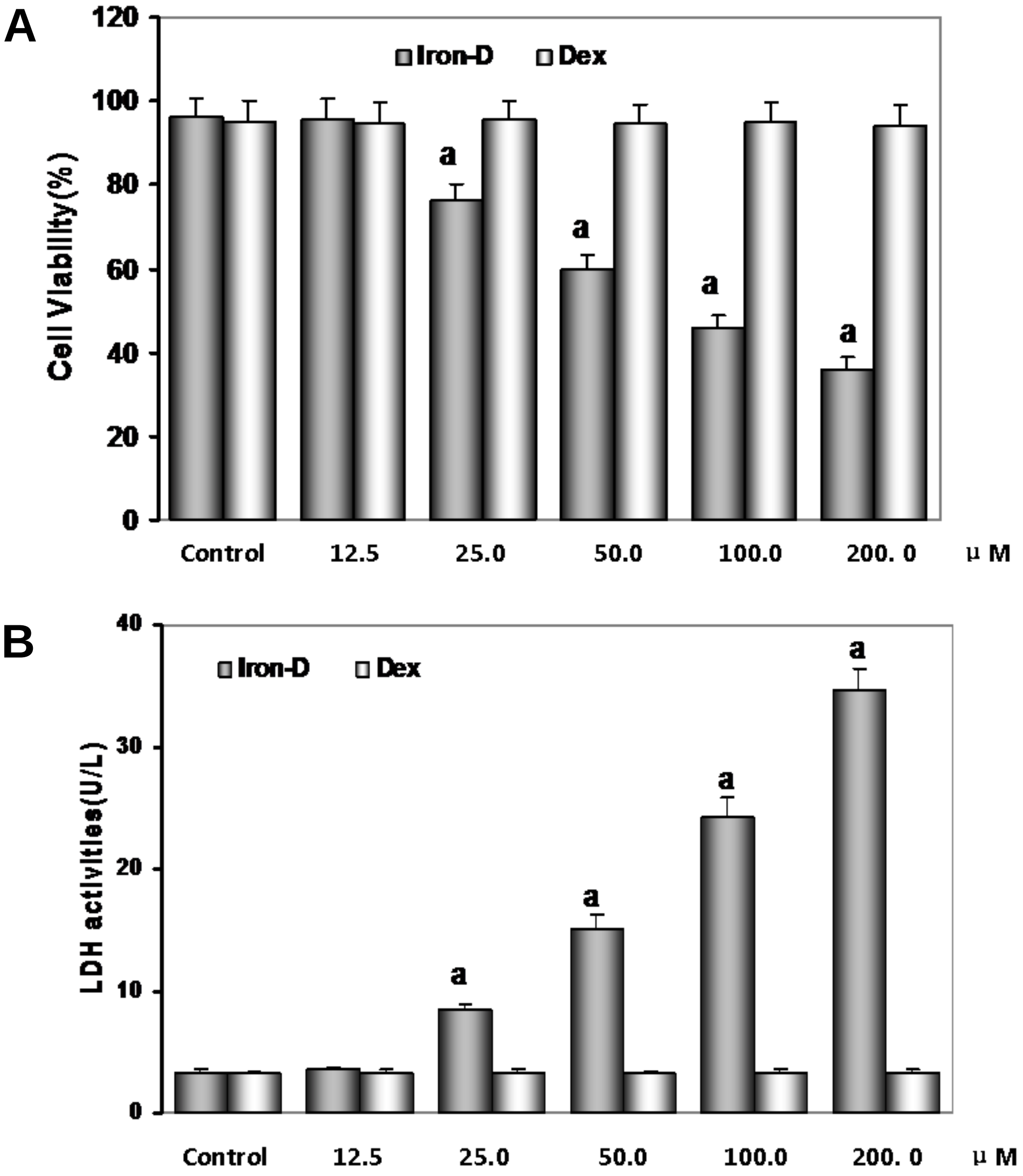
A
120
100
80
60
40
20
0
Iron-D
Dex
Cell Viability(%)
a
a
a
a
Control
12.5
25.0
50.0
100.0
200. 0
μ M
B
40
30
20
10
0
Iron-D
Dex
LDH activities(U/L)
a
a
a
a
Control
12.5
25.0
50.0
100.0
200. 0
μ M

Figure S3

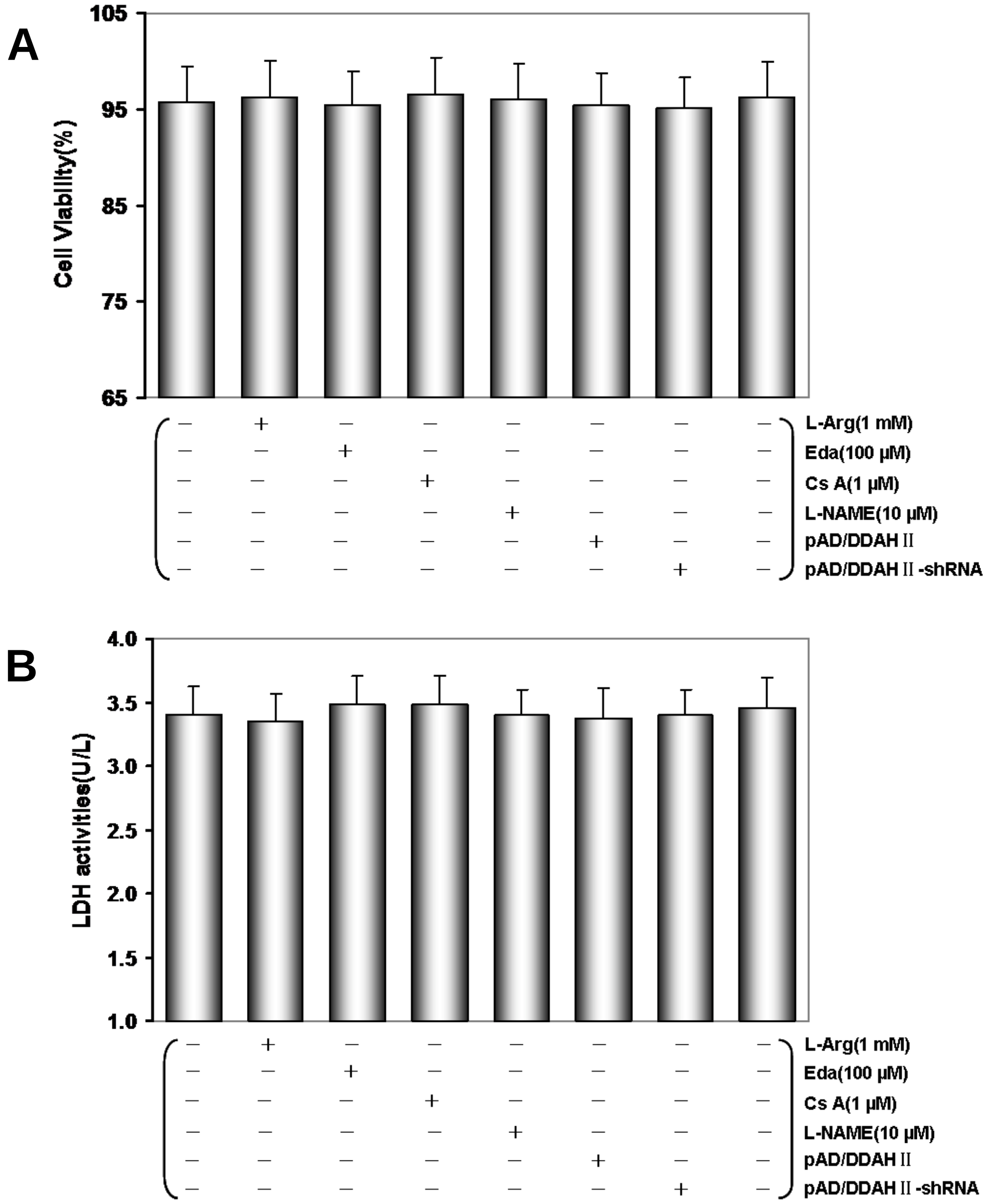
A
B
Cell Viability(%)
105
95
85
75
65
LDH activities(U/L)
4.0
3.5
3.0
2.5
2.0
1.5
1.0
L-Arg(1 mM)
Eda(100 μM)
Cs A(1 μM)
L-NAME(10 μM)
pAD/DDAH II
pAD/DDAH II -shRNA

Figure S4

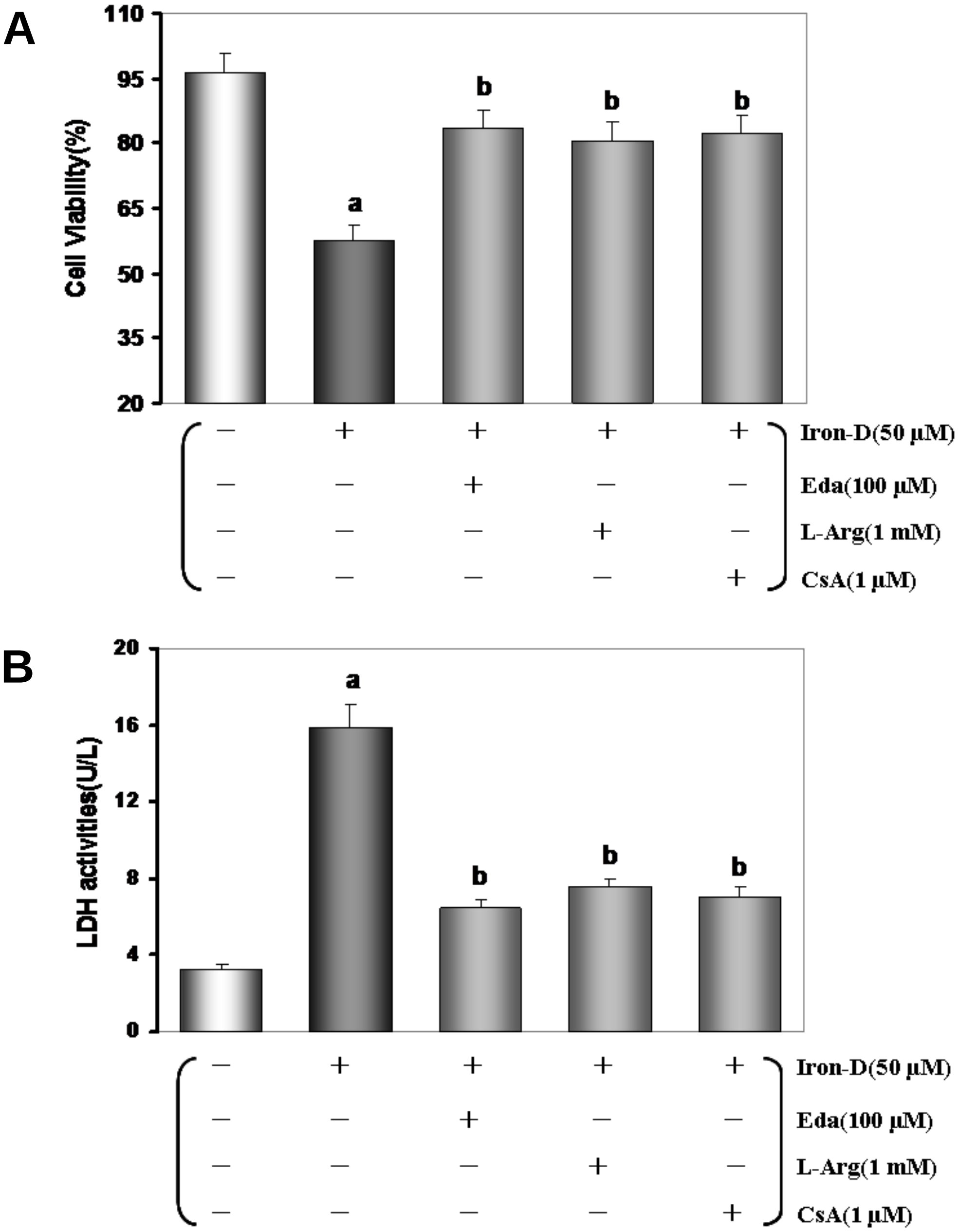
A
Cell Viability(%)
110
95
80
65
50
35
20
a
b
b
b
Iron-D(50 µM)
Eda(100 µM)
L-Arg(1 mM)
CsA(1 µM)
B
LDH activities(U/L)
20
16
12
8
4
0
a
b
b
b
Iron-D(50 µM)
Eda(100 µM)
L-Arg(1 mM)
CsA(1 µM)

Figure S5

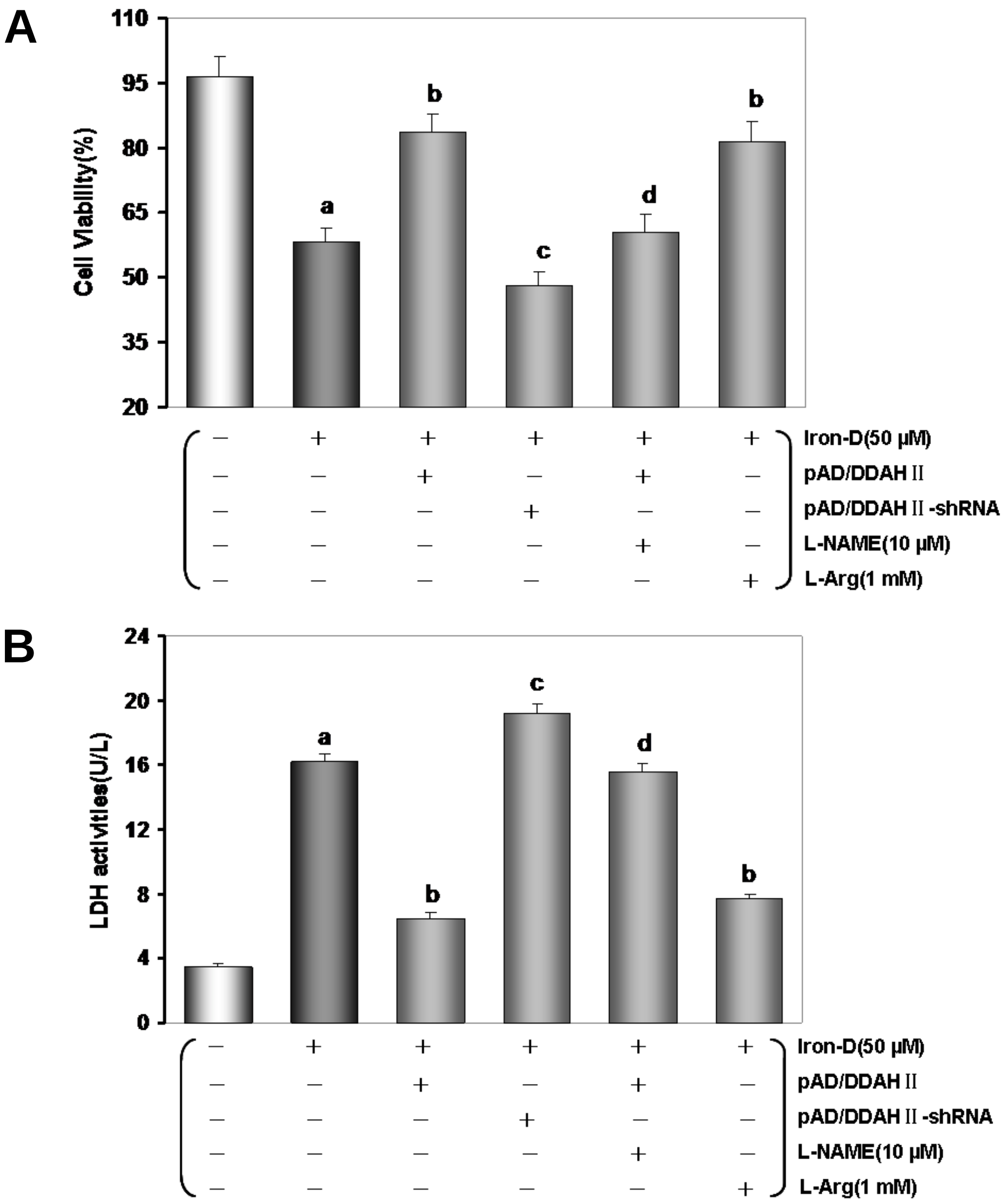
A
110
95
80
65
50
35
20
Cell Viability(%)
a
b
c
d
b
Iron-D(50 μM)
pAD/DDAH II
pAD/DDAH II -shRNA
L-NAME(10 μM)
L-Arg(1 mM)
B
24
20
16
12
8
4
0
LDH activities(U/L)
a
b
c
d
b
Iron-D(50 μM)
pAD/DDAH II
pAD/DDAH II -shRNA
L-NAME(10 μM)
L-Arg(1 mM)

Figure S6
